# Editorial: Fragment-based electronic structure methods for solids

**DOI:** 10.3389/fchem.2023.1165201

**Published:** 2023-02-23

**Authors:** Oleksandr Loboda, Peter Strizhak, Robert W. Góra, Ctirad Cervinka

**Affiliations:** ^1^ AC2T research GmbH, Wiener Neustadt, Austria; ^2^ L.V. Pysarzhevskii Institute of Physical Chemistry, National Academy of Science of Ukraine, Kyiv, Ukraine; ^3^ Wrocław University of Science and Technology, Wrocław, Poland; ^4^ University of Chemistry and Technology, Prague, Czechia

**Keywords:** DFT, GIPAW, NMR, fragment approach, embedding models, molecular crystals, ionic solids

## Fragment-based electronic structure methods for solids

For many years, the Molecular Mechanical (MM) force field (FF) methods have been classical tools for modeling molecular crystal structures, nanomaterials, and surfaces. Being computationally inexpensive, the FF methods are also extensively used in the modeling of the biological systems. Yet, having a set of approximations and based on many parameters derived from the experimental data, the conventional FF methodss are tending to suffer from deficiencies and contain some restrictions. For example, when the energy difference between crystal polymorphs does not exceed several kJ/mol the FFs fail to sufficiently describe well the hydrogen bonding, strong inter/intramolecular interactions, anisotropic electrostatics, or large dispersion contributions. With the rapid development of polarizable FFs, the improvement in treatment of molecular interactions can be achieved but computational cost increases dramatically. Therefore, the dilemma between computational workload and accuracy has to be resolved.

Nowadays, the majority of electronic structure studies are performed with the aid of Quantum Mechanical (QM) methods, which are based on periodic density functional theory (DFT). It should be noted that calculations involving periodic DFT with either plane-wave or atom-centered Gaussian-type basis sets are quite accurate and do not demand high computational cost, once the dispersion corrections are taken into account. However, the tasks involving crystal structure prediction are susceptible to the high extend to the choice of the dispersion correction and type of applied DFT functional.

An alternative approach of electronic structure methods offers a large group array of fragment-based techniques (Gordon et al., 2012; Richard and Herbert, 2012; Collins and Bettens, 2015; Dolgonos et al., 2018; Loboda et al., 2018). The fragment-based methods exploit the idea of divisiondivide the crystal into clusters or individual molecules. Most of the fragment methods utilize the embedding scheme, which combines the treatment of inter-molecular interactions of clusters on a high QM-level with a low-level computation of the entire periodic structure. It is worthwhile to mention that the fragment methods may have two kinds of embedding models: the pure QM:QM and the QM:MM hybrid. If one considers the first model, the Hartree-Fock (HF) and post-HF methods like Second-Order Møller–Plesset Perturbation Theory (MP2) can be used at the high-level (Hofierka and Klimes̆, 2021). In some cases, even coupled cluster methods such as CCSD(T) are feasible for the high-level calculations. As for the lower-tier method, the dispersion-corrected density functional theory (DFT-D) (Grimme, 2011; Grimme et al., 2016) is often the choice of usechosen. Meanwhile, QM:MM model pairs the QM approach for the high-level method and classical MM for low-level periodic calculations.

However, there are some drawbacks that are inevitable upon fragment partitioning. Fragmentation destroys the hydrogen bond cooperativity and impacts the dispersion and induction of many-body interactions. To circumvent this deficiency, the DFT embedding scheme needs to be introduced to mimic interaction between the fragments. The embedding formalism is non-trivial, since it implies an embedding potential common to all subsystems, and therefore it reflects the influence of the environment of the system’s nearest neighbors.

This Research Topic of articles will accustom readers with the leading-edge advances in the performance and development of fragment-based methods. Topics covered in this issue encompass a variety of QM-based methods, such as time-dependent density functionals (TD-DFT), embedded-cluster models, and many-body molecular interactions in molecular solids.

In the first paper by Hartman et al., a molecular correction (MC) approach to the gauge-including plane augmented wave (GIPAW) method is used to calculate electric field gradients (EGF) in molecular solids. It combines the benefits of periodic calculations and single-molecule techniques in the context of electric field gradients (EFG) tensors in molecular solids, which are used to derive nuclear quadrupolar coupling constants and anisotropy parameters. Applying molecular corrections to periodic DFT calculations of NMR parameters of molecular crystals, the authors benchmark the performance of several basis sets and DFT functionals on a relatively large set of crystals, which is important for a sufficient statistical significance of the results. The results of an extensive set of calculations show a noticeable improvement in the calculated NMR observables by including the molecular correction, with minor changes relative to basis set.

In the second paper, Shen et al. tested the performance of another fragment-based method - the electrostatically embedded generalized molecular fractionation with conjugate caps (EE-GMFCC). The efficiency of the EE-GMFCC scheme has been proven for the excited state description of the fluorescent RNA aptamer systems. Their results evidenced that EE-GMFCC calculations, which are one order of magnitude faster, are in excellent agreement with the properties of excited state obtained by traditional full-system time-dependent *ab initio* calculations for so complicated systems. That allowed to describe properties of different fluorescent RNA-aptamer systems.

In the third study, Larsson and Veryazov present the convergence of the electron density with regard to the size of the embedded clusters of MgO and Ni-doped MgO ionic crystals. The main focus of the paper is on the quality of an embedding protocol for ionic oxides and comparison of periodic and cluster calculations.

The branch of fragment-based electronic structure methods invoked to treat solid-state materials is very large and diverse, and this special research selection elucidates only a small part number of manifold applications and recent developments in this Research Topic. Nevertheless, we hope that the selected papers will contribute to further development of fragment-based methods and lead to relevant insights into the balance between accuracy and computational cost, serving as a valuable benchmark for the embedded approaches.
